# Antimicrobial, antioxidant activities, and total phenolic contents of *Pycnanthus angolensis* Sap and *Cryptolepis sanguinolenta* root extracts

**DOI:** 10.1186/s12906-023-04006-8

**Published:** 2023-06-21

**Authors:** Francis Adu-Amankwaah, Hephzibah Sam, Chris Yaw Asare, Felix Charles Mills-Robertson

**Affiliations:** 1 Division of Molecular Biology and Human Genetics, Faculty of Medicine and Health Sciences, DSI-NRF Centre of Excellence for Biomedical Tuberculosis Research: South African Medicinal Research Council Centre for Tuberculosis Research, Stellenbosch University, Cape Town, South Africa; 2 CuroGens Ghana Limited, Accra, Ghana; 3 Biochemistry and Biotechnology Department, Kwame Nkrumah University of Science and Technology, Kumasi, Ghana

**Keywords:** *Pycnanthus angolensis*, *Cryptolepis sanguinolenta*, Aqueous extracts, Ethanol extracts, Chloramphenicol, Tetracycline, Antimicrobial, Total phenolic contents, Antioxidant activity

## Abstract

The death of many people in tropical countries can be attributed to microbial infection, probably, because synthetic antibiotics are failing in the treatment of most microbial infections, attributed to the ability of the microorganisms to mutate and adapt to harsh conditions. This study evaluated, in vitro, the antimicrobial activities, antioxidant potentials, and the total phenolic as well as phytochemical contents of aqueous and ethanol extracts of the root of *Cryptolepis sanguinolenta* (Lindl.) and the crude sap of *Pycnanthus angolensis* (Welw) using selected standard bacteria strains (*Staphylococcus aureus* (ATCC 25,923), *Staphylococcus saprophyticus* (ATCC 15,305), *Escherichia coli* (ATCC 25,922), *Salmonella typhi* (ATCC 19,430), *Pseudomonas aeruginosa* (ATCC 27,853), and *Proteus mirabilis* (ATCC 49,565). The modified agar well diffusion method was used to evaluate the antimicrobial activities of the plant extracts. Chloramphenicol and tetracycline were used as positive controls. The extracts were screened for specific phytochemicals with total phenolic contents were determined using Folin Ciocalteu reagent test. The phytoconstituents observed were alkaloids, cardiac glycosides, and saponins in both *Cryptolepis sanguinolenta* and *Pycnanthus angolensis*. For the antimicrobial activities, all the test bacteria were susceptible to the crude sap of *Pycnanthus angolensis* except *Proteus mirabilis*. In the case of the *Cryptolepis sanguinolenta*, only *S. aureus* was susceptible to both aqueous and ethanol extracts. The total phenolic content, expressed in g/100 g GAE, recorded values of 55.427 ± 4.248 for the crude sap of *Pycnanthus angolensis*, and 11.642 ± 4.248 and 26.888 ± 4.248 for the aqueous and ethanol extracts of *Cryptolepis sanguinolenta*, respectively. It is concluded that *Cryptolepis sanguinolenta* and *Pycnanthus angolensis* are excellent candidates for further development of antimicrobial agents in the fight against microbial infections given the pressing need for novel efficacious agents.

## Introduction

Humans and animals have coexisted with plants since the beginning of time, using them for food and medicine for thousands of years [1]. According to the World Health Organization [2], the use of traditional medicine in treating diseases is based on health practices, knowledge, and beliefs in incorporating plants, animals, and mineral-based spiritual therapies that are applied solely in the prevention and treatment of diseases and illnesses [2].

Despite the significant advances observed in modern medicine, herbal medications have been used to relieve the symptoms of various diseases [3]. Over the years, the efficacy of synthetic antibiotics in treating microbial infections has been limited owing to the ability of microbes to mutate, adapting to harsh conditions and developing resistance towards antibiotics [3]. The interest in medicinal plants in treating diseases is due to their prolonged use by our forefathers in treating themselves, a term known as “traditional” or “indigenous” medicines [2, 4].

*Pycnanthus angolensis* (*P. angolensis*) is a nutmeg tree species in the Myristicaceae family. In Africa, it is widely known as *“*Ilomba” [5, 6] while Twi and Fante dialects in Ghana call it “Otie”. Among the numerous purposes it serves are; the dried fruits of *P. angolensis* used as spices for soups [4]. The seed yields a reddish-yellow brown fat known as Kombo butter or Angola tallow, used for illumination and soap making [7]. The bark is used in treating skin infections, as a purgative, in cleansing the milk of lactating mothers, and in treating chest and cough pains. In Ghana, the bark is used to treat anaemia while in Côte D’Ivoire, it is used as an antidote to ascites and leprosy [5, 6, 8]. In Congo DR, the bark is used to solve infertility problems and also to treat gonorrhoea and malaria. Antimicrobial and anthelminthic properties of the leaves, stem and roots have also been reported [6]. The leaves also possess antioxidant and anti-inflammatory activities [9] and this has necessitated the need to discover the antimicrobial and antioxidant activities of the crude sap extract of *P. angolensis* [5].

*Cryptolepis sanguinolenta* (*C*. *sanguinolenta*) is the most familiar plant of the species of trees belonging to the family Apocynaceae [10, 11]. It is commonly called “Nibima” in Ghana [11] and serves numerous medicinal purposes, including herbalists’ use in treating fever, urinary tract, and upper respiratory tract infections [12, 13]. The most well-known use of *C. sanguinolenta* roots is in the treatment of malaria [14, 15]. It is also historically used to treat insomnia, although the mechanism underlying this has yet to be fully understood [16]. Despite the prevalent use of *P. angolensis* and *C. sanguinolenta* since time immemorial, much knowledge has yet to be found in the literature evaluating their antimicrobial and antioxidative properties [6, 10, 17, 18]. The current study, therefore, seeks to evaluate in vitro the antimicrobial, phytoconstituents, total phenols, and antioxidant activity of the crude sap of *P. angolensis* and the ethanol and aqueous extracts of roots of *C. sanguinolenta.*

## Materials and methods

### Crude plants collection

*P. angolensis* was obtained at Forig, on the Okodee road, Bungalow number 15, Kwame Nkrumah University of Science and Technology (KNUST) in Kumasi (Ashanti Region, Ghana), and the root of *C. sanguinolenta* (specimen voucher number: CSRPM 1911) was obtained at the Centre for Plant Medicine Research (CPMR) in Akuapem-Mampong, Ghana.

Plants’ samples were authenticated by Mr Papa Kofi, a botanist at KNUST, and deposited to the herbarium of the CPMR, at Akuapem-Mampong. The sap of *P. angolensis* was obtained by cutting or making a 45-degree angle in the stem of the tree plant, and the sap was collected using a sterilized 50 mL centrifuge tube. The crude sap was freeze-dried and stored at 4 ^o^C for further downstream analysis.

The fresh roots of *C. sanguinolenta* were washed 2–3 times for 10 min with running tap water and once with sterile water, chopped into small pieces and shade-dried at room temperature until they were dried. The roots were pounded to fine powder and stored in airtight bags for further downstream analysis [11].

### Ethanol extract preparation of the root of *Cryptolepis sanguinolenta*

A mass of 700 g of dried powdered *C. sanguinolenta* roots was taken and soaked in 7 L of 70% ethanol at room temperature for 48 h. The resulting extract was filtered through a two-fold muslin cloth followed by Whatman No. 1 paper, the filtrate was condensed at 65 ^o^C using a rotary evaporator (Buchi Rotavapor R-100, Switzerland), and freeze-dried. The freeze-dried material was stored at 4 ^o^C in the refrigerator for further downstream analysis [19].

### Aqueous extract preparation of the root of *Cryptolepis sanguinolenta*

The powdered root (800 g) was first soaked in distilled water (8 L) for 30 min, followed by 30 min of boiling (100 ^o^C) to produce the aqueous extract. The boiled mixture was allowed to simmer for 30 min to evaporate the excess water. The remaining extract was then freeze-dried for further downstream analysis [19].

### Utilised test microorganisms

The test organisms utilised in this study came from the Komfo Anokye Teaching Hospital (KATH), Kumasi, Ghana. Biochemical and Analytical Profile Index (API) assays were used to confirm the bacteria strains. *Staphylococcus aureus* (ATCC 25,923), *Staphylococcus saprophyticus* (ATCC 15,305), *Proteus mirabilis* (ATCC 49,565), *Pseudomonas aeruginosa* (ATCC 27,853), *Escherichia coli* (ATCC 25,922), and *Salmonella typhi* (ATCC 19,430) were the standard microbes used.

### Inoculum preparation

The National Committee for Clinical Laboratory Standards (2003) technique for inoculum production was slightly modified to prepare the bacterial culture for this study [20]. Stock cultures of the test bacterial strains, *S. aureus* (ATCC 25,923), *S. saprophyticus* (ATCC 15,305) *E. coli* (ATCC 25,922), *S. typhi* (ATCC 19,430), *P. aeruginosa* (ATCC 27,853), and *P. mirabilis* (ATCC 49,565) were streaked onto fresh nutrient agar plates and incubated to obtain isolated colonies. Four to five well-isolated colonies were transferred with an inoculating loop into 5 mL Mueller-Hinton broth and incubated at 37 ^o^C for 16–24 h until turbid. The turbidity was adjusted by adding sterile broth to attain the 0.5 McFarland turbidity standard (McFarland 0.5 equals approximately 10^8^ CFU/mL) [20].

### Antimicrobial assay

The National Committee for Clinical Laboratory Standards (2003) [20] method of the agar well diffusion method was used to determine the antimicrobial property of the sap and root extracts [15]. A sterile cotton swab was dipped into the suspension within 15 min after adjusting the turbidity of the inoculum suspension. Pressing firmly against the inside wall of the tube just above the fluid level, the swab was rotated to remove excess liquid. The swab was streaked over the entire surface of the Mueller-Hinton agar three times, rotating the plate approximately 60 degrees after each application to ensure an even distribution of the inoculum and allowed to dry. A sterile cork borer was used to make holes in the dried medium. All crude extracts were dissolved or resuspended in 20% v/v dimethyl sulfoxide (DMSO) to a concentration of 300 mg/mL. Approximately, 100 µL of each extract was micro-pipetted into the wells/holes labelled. Standard drugs, 30 µg/mL chloramphenicol and tetracycline were placed in respective wells/holes as a positive control for bacteria while 100 µL of 20% v/v of DMSO was used as a negative control. Three other replica plates were for each plate of a particular test microbe and incubated at 35 ^o^C for 24 h. After incubation, the diameters of the zones of inhibition were measured with a sterilized ruler [20].

### Solubility tests

An appreciable amount of the various extracts was each treated with water, absolute ethanol, and 50% ethanol, respectively, and vortexed for a few minutes to qualitatively determine which solvent the various samples are best soluble in.

### Determination of total phenol content

The total phenol content of the extracts was assessed using a modified Gustafson et al. (2012) [21]technique and the Folin-Ciocalteu reagent. A concentration of 5 mg/mL of pyro-gallic acid solution which was diluted to 10 different concentrations using a two-fold dilution served as the reference standard for the analysis. A mass of 5 mg of the extracts was treated with 1 mL of solvent obtained from their respective solubilities. The individual solutions were then diluted to three different concentrations using a two-fold dilution. For the assay, 10 µL of pyro-gallic acid solution and extract solutions prepared in triplicate were treated with 1 µL of distilled water in a 24-well plate. A 50 µL of Folin-Ciocalteu reagent (Folin: Methanol, 1:1, v/v) was added to each dilution. The samples were wrapped in foil and incubated in the dark for five minutes, after which 150 µL of Na_2_CO_3_ was added to the respective dilutions and incubated again at room temperature for two hours. Absorbance was then measured using a microplate spectrophotometer (Tecan Infinite M200 Pro Plate Reader, Austria) at 750 nm against a blank comprised of the various solvents and reagents minus the extracts (standard). The mean value absorbance of the standard was then obtained, and a calibration line was drawn. The concentration of phenolic content in the various extracts was then extrapolated from the graph [21].

### Determination of antioxidant activity

The free radical scavenging activity of the extracts based on the scavenging activity of the stable DPPH free radical was determined by the method described by Sochor et al. [22] with modifications. A concentration of 1 mg/mL of BHT solution (prepared by diluting 1 mg of BHT powder with 1 mL methanol) served as the standard for the analysis. A 0.5 mM DPPH solution was prepared by dissolving 5 mg of DPPH powder in 25 mL of methanol. A mass of 40 mg of the individual extracts was treated with 1 mL of the solvent obtained from their respective solubility tests. The stock solutions were then diluted to 7 different concentrations using a three-fold dilution. The standard stock was also diluted to 7 different concentrations using a two-fold dilution. A colour control (made up of the sample and methanol only but not DPPH) was also carried out for both the standard and the test extracts. The colour control for the extracts was prepared in a three-fold dilution to obtain seven different concentrations.

In contrast, the control for BHT was prepared in a two-fold dilution to obtain seven different concentrations. For the assay, a volume of 100 µL of each extracts and standard concentrations was treated with 100 µL of DPPH solution in a 96-well plate, shaken vigorously, wrapped in foil, and incubated in the dark at room temperature for 20 min, after which the absorbances of all the contents of the plate were read using a microplate spectrophotometer (Tecan Infinite M200 Pro Plate Reader, Austria) at 517 nm of wavelength against their respective blank solutions. All experiments were performed in triplicates and the percentage scavenging activity of the DPPH-free radical was calculated using the formula:

$$\%\;\mathrm{Scavenging}\;\mathrm{activity}=\;\frac{\mathrm{Absorbance}\;\left(\mathrm{control}\right)\;-\;\mathrm{Absorbance}\;\left(\mathrm{sample}\right)\;\times\;100}{\mathrm{Absorbance}\;\left(\mathrm{Control}\right)}$$

Where Absorbance (control) = blank– control.

Absorbance (sample) = mean absorbance of triplicate– control.

A graph of mean percentage antioxidant activity against the concentrations of the various extracts was then plotted and the IC_50_ was calculated using a gradient [22].

### Phytochemical screening

The phytochemical analysis was performed on both aqueous and ethanol extracts of the roots and crude sap using the standard procedures of Edeoga et al. [23]. The foam test for saponins, Wagner’s test for alkaloids, Braymer’s test for tannins, the alkaline reagent and sodium hydroxide test for flavonoids, the ferric chloride test for phenols, Keller Kelliani’s test for cardiac glycosides and Salkowski’s test for terpenoids were carried out [23].

#### Test for alkaloids (Wagner’s test)

The crude sap and root extracts were treated separately with 3.5 drops of Wagner’s reagents (1.27 g of iodine and 2 g of potassium iodide in 100 mL of water). The formation of a reddish-brown inference indicated the presence of alkaloids in crude extracts.

#### Test for saponins (foam test)

A gram of the sap and the root extracts were placed in different test tubes and 2.5 mL of distilled water was added. The mixtures were boiled and filtered. The filtrate was mixed with 3 mL of distilled water and vigorously shaken for about 5 min The Formation of a persistent froth indicated the presence of saponins in the plant sample.

#### Test for terpenoids (Salkowki’s tests)

A volume of 1 mL of chloroform was added to 1 mL of the crude sap and root extracts, followed by a few drops of concentrated Sulphuric acid (H_2_SO_4_). The sample was observed for a reddish-brown colouration to draw an interface that indicates the presence of terpenoids.

#### Test for tannins (Braymer’s test)

A volume of 1 mL of the sap and root extracts was treated with a 10% alcoholic ferric chloride solution. The formation of brownish green or a blue-black colouration showed the presence of tannins.

#### Test for flavonoids (alkaline reagents and sodium hydroxide test)

A volume of 1 mL of the sap and root extracts was treated with drops of a 20% sodium hydroxide (NaOH) solution. The Formation of a persistent froth indicated the presence of saponins in the plant sample.

#### Test for Phenols (the ferric chloride test)

A 5% aqueous ferric chloride was used to treat a portion of the crude sap and root extracts and the.

formation of a deep blue or black colouration indicated the presence of phenols.

#### Test for Cardiac Glycosides (Keller Kelliani’s test)

In test tubes, 1 mL of glacial acetic acid was added to 2.5 mL of sap and root extracts, followed by a drop of ferric chlorides. A volume of 1 mL of concentrated Sulphuric acid (H_2_SO_4_) was added and the formation of a brown ring at the interface indicated the presence of deoxy-sugar characteristic of cardenolides [23].

### Statistical analysis

All grouped data were statistically analysed using Microsoft Excel 2010 and Graph Pad Prism version 8. One-way ANOVA was used for the hypothesis testing where a *p*-value of less than 0.05 was considered to indicate statistical significance. A Bonferroni post-hoc test was used to as a follow-up to the ANOVA to determine which pairwise comparison of means contributed to the overall significant difference that was observed.

## Results

### In vitro antibacterial assay

Tables 1, 2 and 3 show the in vitro antimicrobial screening derived from crude extracts of *P. angolensis* sap and *C. sanguinolenta* root. The *P angolensis* inhibited the growth of all the test bacteria except *P. mirabilis*, while *C. sanguinolenta* could not inhibit any test bacteria except *S. aureus*. Table 4 represents chloramphenicol and tetracycline (standard antibiotics), and Table 5 describes the antibacterial activities of 20% v/v DMSO (a negative control). Chloramphenicol and tetracycline showed activity against all the bacteria tested. Overall, the standard antibiotics showed the highest antibiotic activities as indicated in Table 4.

**Table 1:** Susceptibility of the test microbes to the crude sap of *P. angolensis*

Microorganism	Zones of inhibition (mm)		
	100 mg/mL	200 mg/mL	300 mg/mL
***S. typhi***	10.00 ± 0.00	12.00 ± 0.00	14.50 ± 0.50
*** S. saprophyticus***	10.00 ± 0.00	10.00 ± 0.00	14.00 ± 0.00
*** S. aureus***	10.50 ± 0.50	12.50 ± 1.50	14.50 ± 0.50
***P. aeuroginosa***	10.50 ± 0.50	12.50 ± 0.50	15.00 ± 1.00
***P. mirabilis***	NS	NS	NS
***E. coli***	10.50 ± 0.50	13.50 ± 0.50	15.00 ± 0.00

The antimicrobial activity screening of aqueous and ethanolic extracts of *C. sanguinolenta* gave a 12.00% and 13.5% susceptibility, respectively at 100 mg/mL with only *S. aureus* being the only microbe which showed some level of susceptibility.

**Table 2:** Susceptibility of the test microbes to the ethanol extracts of *C. sanguinolenta*

Microorganism	Zones of inhibition (mm)		
	100 mg/mL	200 mg/mL	300 mg/mL
***S. typhi***	NS	NS	NS
***S. saprophyticus***	NS	NS	NS
***S. aureus***	13.50 ± 0.50	14.00 ± 0.00	15.50 ± 0.50
***P. aeuroginosa***	NS	NS	NS
***P. mirabilis***	NS	NS	NS
***E. coli***	NS	NS	NS

**Table 3:** Susceptibility of the test microbes to the aqueous extracts of *C. sanguinolenta*

Microorganism	Zones of inhibition (mm)		
	100 mg/mL	200 mg/mL	300 mg/mL
***S. typhi***	NS	NS	NS
***S. saprophyticus***	NS	NS	NS
***S. aureus***	12.00 ± 0.00	16.00 ± 0.00	20.00 ± 0.00
***P. aeuroginosa***	NS	NS	NS
***P. mirabilis***	NS	NS	NS
***E. coli***	NS	NS	NS

The in vitro antibacterial screening results obtained from tetracycline and chloramphenicol showed that all the test microbes were susceptible to the antibiotics with *P. mirabilis* showing the least susceptibility to tetracycline (39.00 ± 0.00) and *S. aureus* (48.00 ± 0.00) showing the highest resistance to tetracycline (Table 4). For the chloramphenicol, *S. typhi* (15.00 ± 0.00) showed the lowest susceptibility while *P. aeruginosa* (33.00 ± 0.00) showed the highest resistance.

**Fig. 1:**
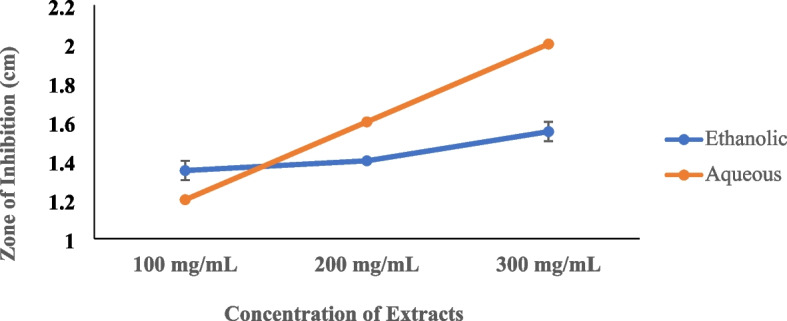
Concentrations of extracts of *C. sanguinolenta* against *S. aureus*

The potency of the concentrations of the extracts against *S. aureus* showed a trend with a positive gradient (Fig. 1). The activity increased with increasing concentration thus, the highest concentration (300 mg/mL) exhibited the highest antimicrobial activity.

**Table 4:** Susceptibility of test microbes to the tetracycline and chloramphenicol

Micro-organisms	Zones of inhibition (mm)	
	Tetracycline	Chloramphenicol
***S. typhi***	44.00 ± 0.00	15.00 ± 0.00
*** S. saprophyticus***	47.00 ± 0.00	26.00 ± 0.00
*** S. aureus***	48.00 ± 0.00	16.00 ± 0.00
***P. aeuroginosa***	45.00 ± 0.00	33.00 ± 0.00
***P. mirabilis***	39.00 ± 0.00	20.00 ± 0.00
***E. coli***	41.00 ± 0.00	21.00 ± 0.00

Dimethyl sulfoxide (DMSO) was used as the negative control in the antimicrobial screening test. The in vitro antimicrobial screening to DMSO showed that there was no susceptibility to any of the microbes (Table 5).

**Table 5:** Susceptibility of the test microbes to the 20% v/v DMSO

Microorganism	Zone of inhibition (mm)
	20% v/v DMSO
***S. typhi***	NS
***S. saprophyticus***	NS
***S. aureus***	NS
***P. aeuroginosa***	NS
***P. mirabilis***	NS
***E. coli***	NS

### Solubility tests

The solubility tests revealed that the crude sap of *P. angolensis* was soluble in 50% ethanol. The aqueous extract of *C. sanguinolenta* was soluble in water and the ethanol form of *C. sanguinolenta* is soluble in 50% ethanol (Table 6).

**Table 6:** Solubility status of the various extracts

Plant extract	Solubility status
***P. angolensis*** **(sap)**	Soluble in a 50:50 ratio of ethanol to water
***C. sanguinolenta*** **(aqueous)**	Soluble in water
***C. sanguinolenta*** **(ethanol)**	Soluble in a 50:50 ratio of ethanol to water

### Total phenol content

**Fig. 2:**
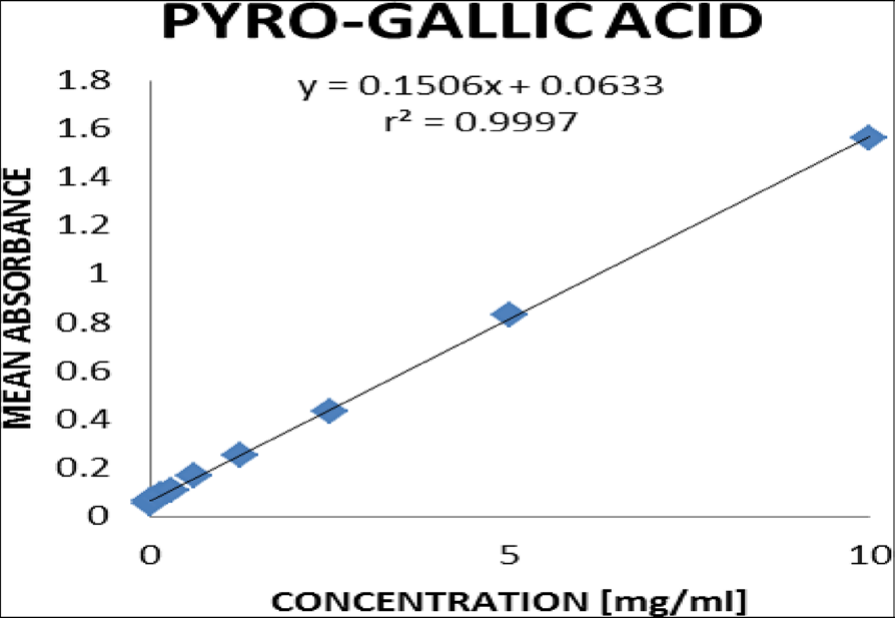
Standard curve of mean absorbances against the concentration of pyro-gallic acid

The plot of mean absorbances against the final concentrations indicated the standard curve of pyro-gallic acid. Based on Fig. 2, the total phenol concentrations present in the various extracts were extrapolated using their absorbances.

The concentration-mean absorbance calibration for the ten stock concentrations of the standard pyro-gallic acid (Fig. 2) revealed that the mean absorbances of the standard were in the range of 0.065 to 1.562 for the concentration range of 0.020 mg/mL to 10 mg/mL. From this study, *P. angolensis* (sap), aqueous *C. sanguinolenta*, and ethanol extract of *C. sanguinolenta* recorded values of 55.427 ± 4.248 SEM, 11.642 ± 4.248 SEM, and 26.888 ± 4.248 SEM, respectively (Table 7). The *p*-value of 0.0001, which was obtained, indicated a statistical difference.

**Table 7:** Total Phenolic Content (TPC) of the various extracts

Samples	TPC (g/100 g GAE ± SEM	*p*-value
*P. angolensis* (sap)	55.427 **±** 4.248	***p*** ** = 0.0001**
* C. sanguinolenta*(aqueous)	11.642 **±** 4.248	
* C. sanguinolenta*(ethanol)	26.888 **±** 4.248	

### Antioxidant activity

**Fig. 3:**
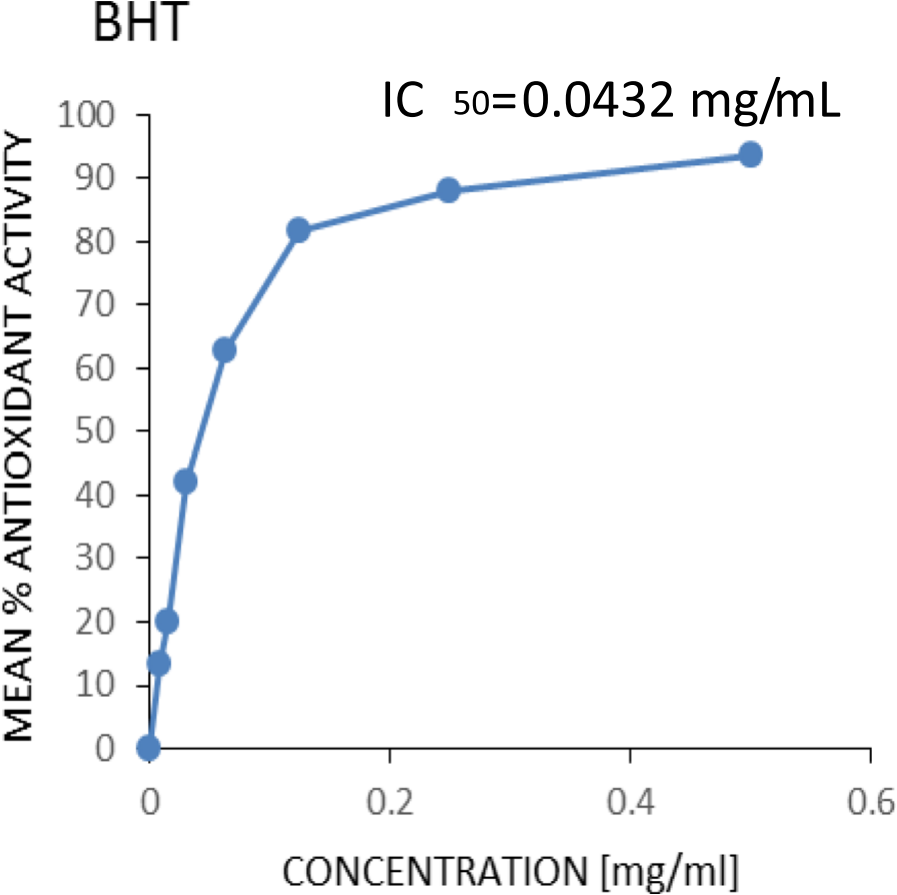
Antioxidant activity of BHT

**Fig. 4:**
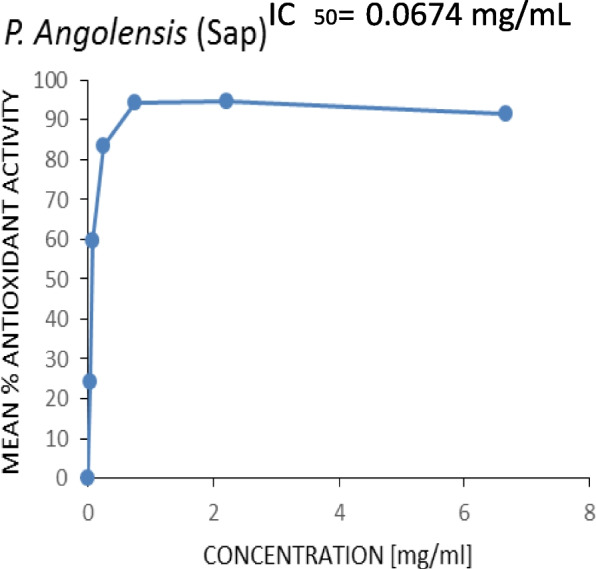
Antioxidant activity of *P. angolensis* (sap)

**Fig. 5:**
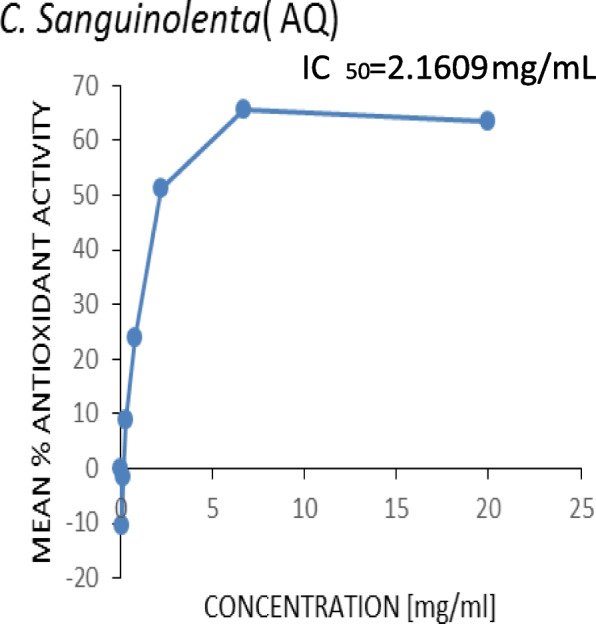
Antioxidant activity of *C*. *sanguinolenta* (aqueous)

**Fig. 6:**
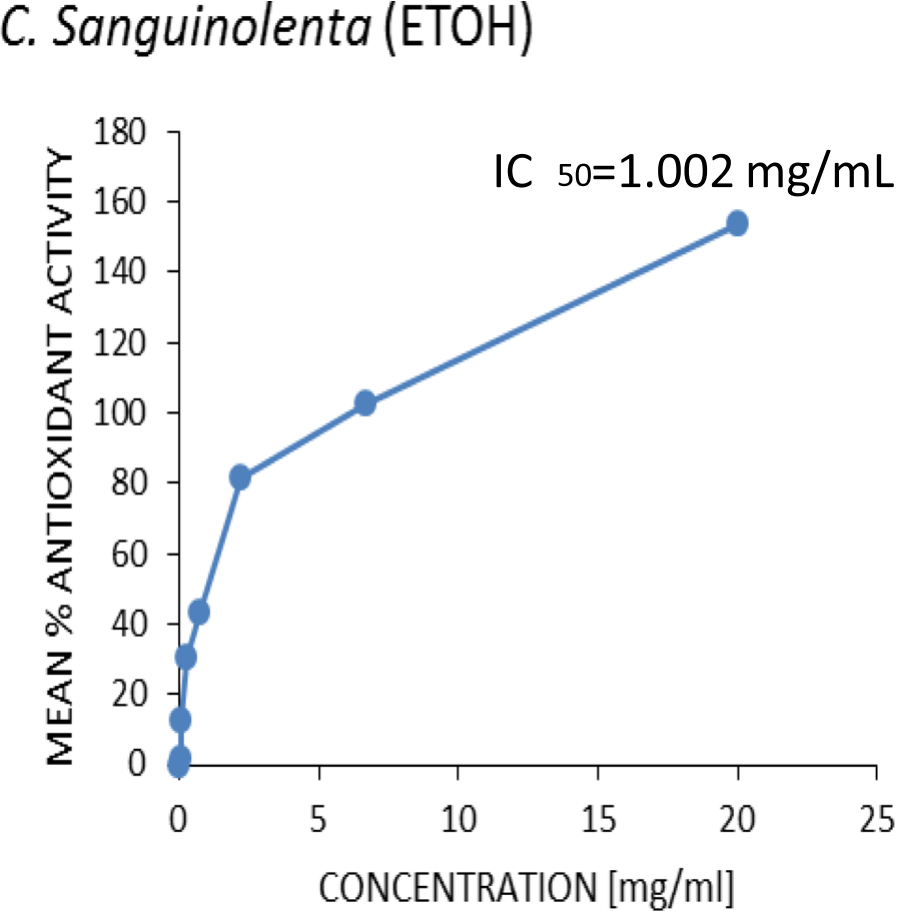
Antioxidant activity of *C. sanguinolenta* (ethanol)

Figures 3, 4, 5 and 6 show the plots of the percentage scavenging activity of the various extracts as well as the standard against their respective concentrations and the respective IC_50_ values obtained. It was observed that the *P. angolensis* (sap) recorded a value of 0.0674 mg/mL which was closest to the value of the BHT standard (0.0432 mg/mL). *C. sanguinolenta* (ethanol) and *C. sanguinolenta* (aqueous) recorded IC_50_ values of 1.002 mg/mL and 2.1609 mg/mL, respectively.

### Phytoconstituents

The phytochemical screening results are represented in Table 8. Six of the phytoconstituents were identified in *P. angolensis* except for flavonoids that were not identified in the crude extract, while in the case of the aqueous and ethanolic extracts of *C. sanguinolenta*, only alkaloids, cardiac glycosides, and saponins were identified.

**Table 8:** Phytoconstituents of the sap of *P*. *angolensis* and the root of *C*. *sanguinolenta*

Plant	Sap of *P. angolensis*	Aq. extract of *C. sanguinolenta*	EtOH extract of *C. sanguinolenta*
phytochemicals			
Alkaloids	**+**	**+**	**+**
Cardiac glycosides	**+**	**+**	**+**
Tannins	**+**	-	-
Saponins	**+**	**+**	**+**
Flavonoids	-	-	-
Terpenoids	**+**	-	-
Phenols	**+**	-	-

‘-‘ Not detected ‘+’ detected

EtOH = Ethanol

Aq = Aqueous

## Discussion

Antimicrobial Resistance (AMR) is a global health issue that causes significant mortality and morbidity. Multiple drug resistance in Gram-positive and Gram-negative bacteria has made treating common illnesses with standard medicines challenging [24]. The rapid proliferation of multiple drug resistance bacteria, along with a lack of effective medications and suitable preventative measures, has prompted the development of innovative treatment alternatives and alternative antimicrobial treatments that are both less expensive and more effective [24].

Bioactive plant constituents have been used in the treatment of both Gram-positive and Gram-negative bacterial infections [25] for centuries. Several studies in the West African sub-region have reported that *C. sanguinolenta* is an anti-malarial plant [10, 11] and there have been other reports on its antimicrobial properties by researchers such as Boakye-Yiadom, Mills-Robertson et al., and Paulo et al. [10–12, 26].

The current study found that *C. sanguinolenta* was susceptible to *S. aureus* (Gram-positive bacteria) but resistant to Gram-negative organisms such as *P. aeuroginosa*, *S. saprophyticus, P. mirabilis*, and *S. typhi* (Tables 2 and 3). This finding was consistent with a study by Boakye-Yiadom [26] who revealed that less than 50 mg/mL of aqueous extract generates mild antibacterial activity, a finding similar to a study of Paulo et al. [12, 26]. Another study by Ramli et al., [27] studied *Ambrosia maritima* and *Bituminaria bituminosa* plants (plant extracts) from Algeria and found that the butanolic extract of *Bituminaria bituminosa* exhibited remarkable antimicrobial activity against *Staphylococcus aureus* and *Candida albicans*, highlighting their potential as sources of antimicrobial agents [27]. This observation could be due to the action of cryptolepine (a bioactive ingredient in *C. sanguinolenta*) on the bacterial cell wall of both Gram-positive and Gram-negative organisms. Gram-negative bacteria have an outer cell membrane, a lipopolysaccharide with low permeability [28]. Some Gram-negative microorganisms also express resistance to inducible cephalosporins or antibiotic efflux pumps that give them high intrinsic resistance to antibiotics; hence, such could be why the extracts of the *C. sanguinolenta* did not work successfully on the selected Gram-negative organisms [28]. These current findings contradict the conclusions from Mills-Robertson et al. [11], in which the plant extract worked against both Gram-positive and Gram-negative microorganisms used in their studies.

*Pycnanthus angolensis* showed high potency against both selected Gram-negative and Gram-positive organisms except *P. mirabilis* and the potency was concentration and dose dependent. This current study finding is consistent with a similar study by Chukwudozie and Ezeonu [18] who reported that the stem bark of *P. angolensis* showed higher inhibition when tested against Gram-positive and negative bacteria. This research complements study by Spengler et al. [29], on the essential oil of *Juniperus oxycedrus* L. ssp. *macrocarpa*, which showed high antibacterial activity against *Salmonella* spp and Gram-positive bacteria, indicating potential for combating antibiotic and antifungal resistance [29]. In those studies, the ethanol extracts of the plant extracts were more susceptible than the aqueous extracts when used against the selected microorganisms [18]. The resistance of *P. mirabilis* could be due to dose dependency and, therefore, will require a higher dose of the plant extract to be susceptible [24]. The current study shows that the phytochemicals in *P. angolensis* are potent against both Gram-negative and Gram-positive organisms in a dose-dependent pattern. It will be ideal to investigate this further in developing novel antimicrobial agents to tackle the growing threat of AMR [24].

Results obtained from *P. angolensis* has confirmed the early claims by Omobuwajo et al. [9] and Sofidiya and Awolesi [5] (Table 1), that *P. angolensis* is a remedy for chest pains and skin diseases such as boils, furuncles caused by *S. aureus*, wound healing, and gastrointestinal ailment which are usually caused by some of these microorganisms [5, 9]. Agyare et al. and Onocho and Otula [8, 14] also claimed that *P. angolensis* is a medicinal source for the management of food poisoning, bloody diarrhoea, and urinary tract infections caused by *S. typhi* and *S. saprophyticus*. In this current study, we did not evaluate the potential interactions between the drug compounds under investigation specifically, their synergistic, antagonistic, or additive effects. It is important to note that such assessments would require further experimentation to determine the extent to which the drugs interact with one another, and whether their combined effects result in a greater or lesser therapeutic outcome than anticipated based on their individual efficacy. Therefore, while our current findings are informative, they do not provide insights into the potential synergistic, antagonistic, or additive effects of the drugs, and further studies will be required to elucidate these potential interactions.

Phenols have been reported to have antiseptic, anti-inflammatory, antimicrobial, and anti-tumour properties, and tannins have also been reported to have anti-ageing properties as well as skin regeneration, anti-inflammatory and diuretic properties [30]. According to Agyare et al. [14], flavonoids have splendid antimicrobial and anticancer activities, while alkaloids are used as painkiller medications [31]. Phenolic compounds are known to possess potent antioxidant properties and are believed to be the primary contributors to the antioxidant activity of plant extracts [30]. Therefore, it is generally expected that plant extracts with higher total phenolic content will exhibit greater antioxidant activity [30]. Similarly, certain phytochemicals, such as flavonoids, alkaloids, and terpenoids, have been shown to possess antibacterial properties. Therefore, plant extracts that contain high levels of these phytochemicals are more likely to exhibit antibacterial activity [30].

Several studies have investigated the correlation between total phenolic content, phytochemical composition, and antioxidant or antibacterial activity of plant extracts. These studies have generally found a positive correlation between these variables, suggesting that plant extracts with high levels of phenolic compounds and specific phytochemicals tend to exhibit higher antioxidant and antibacterial activity. Krasteva et al. [32] conducted a study to evaluate the phenolic content, composition, antioxidant and antibacterial activities of four grape seed extracts (Cabernet Sauvignon, Marselan, Pinot Noir, and Tamyanka). The extracts exhibited high total phenolic content, with Pinot Noir having the highest antimicrobial activity against *Staphylococcus aureus*, *Bacillus cereus*, and *Escherichia coli* [32, 33]. The extracts’ components were determined using HPLC, and high contents of catechin, epicatechin, and procyanidin B1 were found. The extracts showed high sensitivity to the tested bacteria, and a correlation was found between the phenolic content of the GSEs and their antibacterial potential [32]. Another study by Jawhari et al. [33] evaluated the mineral and chemical compositions, total phenolic and flavonoid contents, and antimicrobial and antioxidant activities of two varieties of *Anacyclus pyrethrum* (L.). The hydroalcoholic extracts from different parts of the plants (leaves, capitula, roots, and seeds) were analyzed [33]. The results revealed both varieties’ exciting mineral and chemical compositions, with specific active compounds detected in each. The antioxidant and antimicrobial activities of the extracts showed promising properties, with leaves, capitula, and seeds exhibiting similar activity patterns [33]. Results from this study (Table 7) revealed that the crude sap of *P. angolensis* contained the highest amount of total phenol content compared to the roots of *C. sanguinolenta*. In comparison, it was observed that the ethanol extract had a significantly higher total phenol content than the aqueous solution. The aqueous solution recorded a low value, possibly due to the inability of water to adequately extract non-polar polyphenols into the solution [34]. In the current study, 70% ethanol was used to prepare the ethanol extract. Ethanol when combined with water, has a much greater potential to extract polar and non-polar polyphenols into solution than when absolute concentration is used [34]. Low values were also recorded in the aqueous extract, possibly due to the action of the enzyme polyphenol oxidase, which works best in an aqueous medium and acts on polyphenols and degrades them, thereby reducing their presence in solution [34]. From the hypothesis test carried out, it was realised that a comparison of the three extracts produced a *p*-value of 0.0001, indicating that the various extracts were very different from each other and, as such, one extract could not be substituted for another for its usage in the manufacturing of potent drugs [35].

This study showed an increase in the mean percentage of antioxidant activity as the concentrations increased. This was reflected in all the extracts and the standard BHT to which the extracts were compared. It was observed that *P. angolensis* (sap), *C. sanguinolenta* (aqueous), and *C. sanguinolenta* (ethanol) recorded IC_50_ values of 0.0674 mg/mL, 2.1609 mg/mL and 1.002 mg/mL, respectively, compared to the BHT of 0.0432 mg/mL (Figs. 3, 4, 5 and 6). Comparing the extracts for the study to the standard, *P. angolensis* (sap) recorded values comparable to the reference value [36]. Even though the IC_50_ values of the aqueous and ethanol forms of *C. sanguinolenta* are not close to that of the standard, it can be said conclusively that they are good antioxidants as few amounts of these extracts can mop up 50% of free radicals [37]. It was also observed that *C. sanguinolenta* (ethanolic) recorded an IC_50_ value much closer to the standard than *C. sanguinolenta* (aqueous). This indicates that the ethanol crude extract of *C. sanguinolenta* is a much better antioxidant than the aqueous extract. This is likely due to the percentage of ethanol (70%) used for the extraction. Coupled with some amount of water, ethanol had a more significant potential to dissolve more phenolic compounds than using only distilled water or ethanol [38]. It must be noted, that the closer an IC_50_ value of an extract is to zero, the more likely it is for the extract to possess potent antioxidant capabilities [21]. Thus, the crude sap of *P*. *angolensis* is a more powerful antioxidant than the ethanol extract of *C. sanguinolenta*, which is also a better antioxidant than the aqueous extract of *C*. *sanguinolenta*. On the whole, all three extracts proved to be very effective antioxidants. The current study findings agree with study by Khanc et al. [39], where it was reported that in the nitric oxide scavenging experiment, the crude extract of *P. angolensis* showed astounding efficacy with a 99.0% Radical Scavenging Activity (RSA) compared to the reference, n-propyl gallate (90.3% RSA) [39]. Another study conducted by Oladimeji and Akpan [6], also showed that *P. angolensis* had a moderate antioxidant activity of 0.55 µg/mL when compared with the standard drug (Vitamin C) with an antioxidant activity of 0.45 µg/mL [6]. Furthermore, the antioxidant activities of *P. angolensis* were better than those of vitamins A and E at 0.57 and 0.59 µg/mL [6], respectively. The antioxidant capabilities of the extracts were instructive since the phytochemical analysis of the plants revealed the presence of terpenes, flavonoids, and tannins, all of which have antioxidant properties. Studies have shown a direct correlation between the total phenol content and extracts’ antioxidant activity [36, 40, 41]. It is therefore not surprising that the crude sap of *P. angolensis*, which recorded a higher IC_50_ value of 0.0674 mg/mL, had a higher amount of phenol content (55.427 ± 4.248) compared to the ethanol extract of *C. sanguinolenta* which also recorded a higher IC_50_ value of 1.002 mg/mL and an amount of 26.888 ± 4.248 g/100 g GAE of phenol content than its aqueous extract which recorded the least values [10, 11].

The present work revealed that the root extracts (aqueous and ethanolic) of *C. sanguinolenta* possess alkaloids, cardiac glycosides, and saponins. In contrast, the crude sap of *P. angolensis* possessed alkaloids, cardiac glycosides, tannins, saponins, terpenoids, and phenols. These secondary metabolites have been found to possess antimicrobial and anticancer properties. Alkaloids are mostly known for their toxicity against cells of foreign organisms, and these have the potential to eliminate and reduce human cancer cell lines [14]. Alkaloids are naturally occurring metabolites in plants and are mostly present as heterocyclic compounds containing nitrogen atoms (which are very essential for plant growth) and are in the form of salts coupled with organic acids [14]. It was therefore not surprising that they were found to be present in various extracts. Eleazu and Eleazu [42] reported that isolated alkaloids and their derivatives possess medicinal properties due to their antispasmodic, antibacterial, and analgesic properties [42].

Tannins are known to form irreversible complexes with proline-rich proteins [43]. Parekh and Chanda [44] also found that tannins react with proteins to produce essential effects for the treatment of inflamed or ulcerated tissues. Plants rich in tannins are astringent and may be used for treating intestinal disorders like dysentery and diarrhoea [44]. *P. angolensis* is a plant used to treat intestinal disorders like dysentery and diarrhoea diseases in West Africa. This may prove the antimicrobial and anticancer properties of *P. angolensis* based on its phytochemical constituents [10]. The absence of flavonoids in both aqueous and ethanolic extracts of the root extracts of *C. sanguinolenta* and crude sap of *P. angolensis* does not mean a lack of the bioactive constituents [11]. However, this may be due to the low levels of the bioactive compounds in the crude plant extracts used in this current study [11]. Saponins possess hypolipidemic and anticancer activities and are also important for co-functioning with cardiac glycosides to enable them to carry out their activities which include serving as cardiac drugs and promoting nitrogen retention in osteoporosis or with animals with wasting illness [45–47]. Terpenoids also have a broad range of properties including antitumor, antiviral, bactericidal, fungicidal, analgesic, anti-inflammatory, spermicidal, and cytotoxic activities [48]. Phenolic compounds are most notable for their antioxidant action due to their high tendency to chelate metals and inactivate their actions [49]. All these medicinal effects of the various phytoconstituents make them possible for their usage in treating numerous diseases.

Results from this current study are consistent with the works carried out by others. Considering the sap of *P. angolensis*, the work done by Udeozo et al. [50], on the powdered stem revealed the presence of flavonoids, alkaloids, saponins, tannins, terpenoids, and glycosides. Oladimeji et al. [6], who also worked on the ethanolic extract revealed the presence of saponins, cardiac glycosides, and terpenoids except for alkaloids, tannins, and flavonoids. Akinyenye and Olatunya [51] also confirmed positive tests for alkaloids, saponins, tannins, terpenoids, flavonoids, and cardiac glycosides upon working on the aqueous extract of the plant. Their results were also compared with that of Udeozo et al. [50]. This study was consistent with the works by Mills-Robertson et al. [11], who worked on the cold and hot water extracts as well as the ethanol extracts and revealed the presence of alkaloids. Bunalema [52], worked on the crude extracts of the roots and revealed the presence of alkaloids, tannins, and flavones. Claude et al. [52] worked on the methanol extracts and obtained positives for alkaloids, tannins, and flavones just as obtained by Bunalema [52]. Mills-Robertson et al. [10], worked on the aqueous, ethanol, and chloroform extracts that revealed the presence of alkaloids and the absence of saponins and flavonoids in all three extracts. Chahar et al. [13], also worked on the aqueous, ethanol, and chloroform extracts and their study revealed the presence of alkaloids and terpenes (for only the aqueous extract) and the absence of saponins and flavonoids in all the three extracts. A study carried out by Chime [53] on the ethanol crude extracts of *C. sanguinolenta* revealed the presence of alkaloids, terpenoids and glycosides. Saponins, tannins, but flavonoids were absent. From the various studies on *C. sanguinolenta*, it was realized that alkaloids tested positive throughout and this confirms the work done by Gibbons et al. [54], who not only identified the alkaloid cryptolepine but also went a step further to isolate this potent alkaloid. The differences in results from this study as compared to the others could be genuinely due to their absence or the difference in the methods of preparation and the types or parts of crudes used in the various extracts [10].

## Conclusion

This study provides valuable insight into the potential antimicrobial properties and total phenolic content of two selected West African plants, *P. angolensis* and *C. sanguinolenta*. The in vitro results suggest that these plant extracts may have promising applications in the treatment of microbial infections and other non-communicable diseases.

## Data Availability

The datasets used and analysed during the current study are available from the corresponding authors upon reasonable request.

## References

[1] Maqsood S, Singh P, Samoon H, Khansaheb Balange A (2010). International aquatic research effect of dietary chitosan on non-specific immune response and growth of Cyprinus carpio challenged with Aeromonas hydrophila. Int Aquat Res.

[2] World Health Organization. National policy on traditional medicine and regulation of herbal medicines: Report of a WHO global survey. World Health Organization. 2005. ISBN: 9241593237

[3] Bérdy J. Thoughts and facts about antibiotics: Where we are now and where we are heading. J Antibiot (Tokyo). 2012;65(8):385–95. 10.1038/ja.2012.27 22511224

[4] Ong CK, Bodeker G, GRundy C, Burford G. Shein. K. WHO global atlas of traditional, complementary and alternative medicine. World Health Organization, the WHO Centre for Heath Development; 2005.

[5] Sofidiya MO, Awolesi AO. Antinociceptive and antiulcer activities of Pycnanthus angolensis. Revista Brasileira de Farmacognosia. 2015;25(3):252–7.

[6] Oladimeji OH, Ubulom PE, Igboasoiyi AC, Ndukwe K, Nia R (2006). Some biological activities of Pycnanthus angolensis (Welw.) Warb. J Pharm Bioresources.

[7] Abrantes M, Mil-Homens T, Duarte N, Lopes D, Cravo P, Madureira M, et al. Antiplasmodial activity of Lignans and extracts from Pycnanthus angolensis. Planta Med. 2008;31(11):1408–12. 10.1055/s-2008-1081317 18671202

[8] Onocha PA, Otunla EO (2010). Biological activities of extracts of Pycnanthus angolensis (Welw.) Warb. Arch Appl Sci Res.

[9] Omobuwajo OR, Adesanya SA, Babalola GO. Isoflavonoids from Pycnanthus angolensis and Baphia nitida. Phytochemistry. 1992;31(3):1013–4.

[10] Mills-Robertson FC, Tay SCK, Duker-Eshun G, Walana W, Badu K. In vitro antimicrobial activity of ethanolic fractions of Cryptolepis sanguinolenta. Ann Clin Microbiol Antimicrob. 2012;11. 10.1186/1476-0711-11-16. 10.1186/1476-0711-11-16 PMC3473295 22709723

[11] Mills-Robertson FC, Aboagye FA, Duker-Eshun G, Kaminta S, Agbeve S. In vitro antimicrobial activity of Cryptolepis sanguinolenta (periplocaceae). Afr J Pharm Pharmacol. 2009;3(9):476–80. Available from: http://www.academicjournals.org/ajpp.

[12] Paulo A, Duarte A, Gomes ET. In vitro antibacterial screening of Cryptolepis sanguinolenta alkaloids. J Ethnopharmacol. 1994;44(2):127–30. 10.1016/0378-8741(94)90079-5 7853864

[13] Chahar N, Deswal S, Mahalwal VS. In vitro antimicrobial activity of ethanolic fractions of Cryptolepis sanguino-lenta. 2013.

[14] Agyare C, Asase A, Lechtenberg M, Niehues M, Deters A, Hensel A. An ethnopharmacological survey and in vitro confirmation of ethnopharmacological use of medicinal plants used for wound healing in Bosomtwi-Atwima-Kwanwoma area, Ghana. J Ethnopharmacol. 2009;125(3):393–403. 10.1016/j.jep.2009.07.024 19635544

[15] Agyepong IA, Adjei S. Public social policy development and implementation: a case study of the Ghana National Health Insurance scheme. Health Policy Plan. 2008;23(2):150–60. 10.1093/heapol/czn002 18245803

[16] Sofowora A (1996). Medicinal plants and traditional medicine in Africa.

[17] Simic A, Kroepfl D, Simic N, Ogunwande IA. Pycnanthus angolensis (Welw) Excell: Volatile Oil Constituents and Antimicrobial activity. 2006.

[18] Chukwudozie IK, Ezeonu IM. Antimicrobial properties and acute toxicity evaluation of Pycnanthus angolensis stem bark. Sci Afr. 2022;16:e01185.

[19] Mills-Robertson FC, Tay SC, Duker-Eshun G, Walana W, Badu K. In vitro antimicrobial activity of ethanolic fractions of Cryptolepis sanguinolenta. Ann Clin Microbiol Antimicrob. 2012;18(1):16. 10.1186/1476-0711-11-16 PMC3473295 22709723

[20] Kiehlbauch JA, Hannett GE, Salfinger M, Archinal W, Monserrat C, Carlyn C (2000). Use of the National Committee for Clinical Laboratory Standards guidelines for disk diffusion susceptibility testing in New York state laboratories. J Clin Microbiol..

[21] Gustafson K, Giurleo D, Ho C, Dang W, Pan M, Wu Q, & Simon J. Antioxidant, anti-inflammatory and neuroprotective activities of plastoquinones from the seed fat of Pycnanthus Angolensis. Planta Medica. 2012;78(11). 10.1055/s-0032-1320385.

[22] Sochor J, Ryvolova M, Krystofova O, Salas P, Hubalek J, Adam V, Trnkova L, Havel L, Beklova M, Zehnalek J, Provaznik I, Kizek R (2010). Fully Automated Spectrometric Protocols for Determination of Antioxidant Activity: Advantages and Disadvantages. Molecules..

[23] Edeoga HO, Okwu DE, Mbaebie BO. Phytochemical constituents of some nigerian medicinal plants. Afr J Biotechnol. 2005;4(7):685–8.

[24] Frieri M, Kumar K, Boutin A. Antibiotic resistance. J Infect Public Health 2017;10(4):369–78. 10.1016/j.jiph.2016.08.007 27616769

[25] Sawer IK, Berry MI, Ford JL. The killing effect of cryptolepine on Staphylococcus aureus. Lett Appl Microbiol. 2005;40(1):24–9. 10.1111/j.1472-765X.2004.01625.x 15612998

[26] Boakye-Yiadom K. Antimicrobial Properties of some west african Medicinal plants II. Antimicrobial activity of aqueous extracts of Cryptolepis Sanguinolenta (Lindl.) Schlechter. Q J Crude Drug Res 1979;17(2):78–80.

[27] Ramli I, Zerizer S, Gali L, Sakhri FZ, Kabouche Z, Usai D et al. In vitro and in vivo bioactivities of Ambrosia maritima and Bituminaria bituminosa organic extracts from Algeria. J Infect Dev Ctries. 2022;16(6):1064–74. 10.3855/jidc.16788 35797302

[28] Hancock REW. Resistance Mechanisms in Pseudomonas aeruginosa and other nonfermentative gram-negative Bacteria. Clin Infect Dis. 1998;27(s1):93–9. 10.1086/514909 9710677

[29] Spengler G, Gajdács M, Donadu MG, Usai M, Marchetti M, Ferrari M, Mazzarello V, Zanetti S, Nagy F, Kovács R. Evaluation of the Antimicrobial and Antivirulent Potential of Essential Oils Isolated from Juniperus oxycedrus L. ssp. macrocarpa Aerial Parts. Microorganisms. 2022;10(4). 10.3390/microorganisms10040758. 10.3390/microorganisms10040758 PMC9032431 35456809

[30] Doughari JH. Phytochemicals: extraction methods, basic structures and mode of action as potential chemotherapeutic agents. INTECH Open Access Publisher Rijeka, Croatia; 2012.

[31] Cousins D, Huffman MA (2002). Medicinal properties in the diet of gorillas: an ethno-pharmacological evaluation. Afr Study Monogr.

[32] Krasteva D, Ivanov Y, Chengolova Z, Godjevargova T. Antimicrobial Potential, Antioxidant Activity, and Phenolic Content of Grape Seed Extracts from Four Grape Varieties. Microorganisms. 2023;11(2). 10.3390/microorganisms11020395. 10.3390/microorganisms11020395 PMC9963647 36838361

[33] Jawhari FZ, Moussaoui AEL, Bourhia M, Imtara H, Saghrouchni H, Ammor K et al. Anacyclus pyrethrum var. Pyrethrum (l.) and anacyclus pyrethrum var. depressus (ball) maire: Correlation between total phenolic and flavonoid contents with antioxidant and antimicrobial activities of chemically characterized extracts. Plants. 2021;10(1):1–19. 10.3390/plants10010149 PMC7828480 33451098

[34] Koleva II, van Beek TA, Linssen JPH, de Groot A, Evstatieva LN. Screening of plant extracts for antioxidant activity: a comparative study on three testing methods. Phytochem Anal. 2002;13(1):8–17. 10.1002/pca.611 11899609

[35] Adu-Amankwaah F, Tapfuma KI, Hussan RH, Tshililo N, Baatjies L, Masiphephethu MV et al. Cytotoxic activity of Cape Fynbos against triple-negative breast cancer cell line. South Afr J Botany. 2022;150:702–10. Available from: https://linkinghub.elsevier.com/retrieve/pii/S0254629922004239.

[36] Stanojević L, Stanković M, Nikolić V, Nikolić L, Ristić D, Čanadanovic-Brunet J, Tumbas V. Antioxidant Activity and Total Phenolic and Flavonoid Contents of Hieracium pilosella L. Extracts. Sensors. 2009;9(7). 10.3390/s90705702. 10.3390/s90705702 PMC3274148 22346723

[37] Brand-Williams W, Cuvelier ME, Berset C. Use of a free radical method to evaluate antioxidant activity. LWT - Food Science and Technology, 1995;28(1). 10.1016/S0023-6438(95)80008-5.

[38] Martins M, Arantes R, Candeias S, Tinoco F, Cruz-Morais MT. Antioxidant, antimicrobial and toxicological properties of Schinus molle L. essential oils. J Ethnopharmacol. 2014;151(1):485–92. 10.1016/j.jep.2013.10.063 24231069

[39] Khanc IO, Ngandeud F, Pepin EA, Choudharyc IM (2008). Antioxidant activity of the crude extract of the fruits of Pycnanthus angolensis and α-glucosidase inhibitory activity of its constituents. Pharmacologyonline.

[40] Harborne JB (1973). Phenolic Compounds. Phytochemical methods.

[41] Ştefanescu BE, Szabo K, Mocan A, Crisan G. Phenolic compounds from five ericaceae species leaves and their related bioavailability and health benefits. In Molecules. 2019;24,(11). MDPI AG. 10.3390/molecules24112046. 10.3390/molecules24112046 PMC6600139 31146359

[42] Eleazu CO, Eleazu KC (2012). Physico-chemical properties and antioxidant potentials of 6 new varieties of ginger (Zingiber officinale). Am J Food Technol.

[43] Alhaji S (2010). Some medicinal plants of arabian Pennisula. J Med Plants Res.

[44] Georgakilas AG, Redon CE, Ferguson NF, Kryston TB, Parekh P, Dickey JS et al. Systemic DNA damage accumulation under in vivo tumor growth can be inhibited by the antioxidant Tempol. Cancer Lett [Internet]. 2014;353(2):248–57. Available from: https://www.sciencedirect.com/science/article/pii/S0304383514003814. 10.1016/j.canlet.2014.07.030 PMC4167057 25069035

[45] Nahar L, Sarker SD. Chemistry for pharmacy students: general, organic and natural product chemistry. John Wiley & Sons. 2019. ISBN: 1119394430.

[46] Maurya R, Singh G, Yadav PP (2008). Antiosteoporotic agents from natural sources. Stud Nat Prod Chem.

[47] Madziga HA, Sanni S, Sandabe UK (2010). Phytochemical and elemental analysis of Acalypha wilkesiana leaf. J Am Sci.

[48] Patocka J (2003). Biologically active pentacyclic triterpenes and their current medicine signification. J Appl Biomed.

[49] Kessler M, Ubeaud G, Jung L. Anti- and pro-oxidant activity of rutin and quercetin derivatives. J Pharm Pharmacol. 2010;55(1):131–42. 10.1211/002235702559 12625877

[50] Udeozo IP, Eboatu AN, Arinze RU, Okoye HN (2011). Some fire characteristics of fifty-two nigerian Timbers. Anachem J.

[51] Akinyeye RO, Olatunya AM (2014). Phytochemical screening and mineral composition of the bark of some medicinal trees in Ondo State, Nigeria. Med Aromat Plant Res J.

[52] Kirimuhuzya C, Bunalema L, Waako P, Tabuti JRS, Orodho J, Magadula JJ (2012). Efficacy of Cryptolepis sanguinolenta root extract on slow-growing rifampicin resistant Mycobacterium tuberculosis. J Med Plants Res.

[53] Momoh Onyishivi. MA. Formulation and evaluation of ethanolic extract of Cryptolepis sanguinolenta root tablets. Innovare J Ayurvedic Sci. 2014;10–3.

[54] Gibbons S, Fallah F, Wright CW. Cryptolepine hydrochloride: a potent antimycobacterial alkaloid derived from Cryptolepis sanguinolenta. Phytotherapy Research: An International Journal devoted to pharmacological and toxicological evaluation of natural product derivatives. 2003;17(4):434–6. 10.1002/ptr.1284 12722159

